# Tonsillolith

**DOI:** 10.1002/ccr3.4243

**Published:** 2021-06-22

**Authors:** Kiyoshi Shikino, Masatomi Ikusaka

**Affiliations:** ^1^ Department of General Medicine Chiba University Hospital Chiba Japan

**Keywords:** dysphagia, tonsil stone

## Abstract

Small tonsilloliths are usually asymptomatic, whereas large tonsilloliths are sometimes associated with recurrent sore throat and odynophagia.

## Graphical abstract

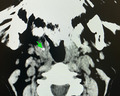

A 68‐year‐old woman developed a sudden right‐sided odynophagia 3 weeks ago. She visited an otolaryngologist after 2 weeks. She was concerned about her halitosis or bad breath and underwent fiber‐optic laryngoscopy, which did not reveal any abnormalities such as foreign bodies. She was prescribed antibacterial drugs, but her symptoms did not improve. Physical examination revealed no tonsillar abnormalities, whereas careful oral cavity palpation showed a firm, nodular mass in the tonsillar crypts. Computed tomography revealed a dense calcified mass in the right palatine tonsil (Figure 1). The mass was excised under local anesthesia and measured 6 mm × 4 mm in size, and appeared hard, and has an irregular surface (Figure 2). The compositional analysis of the stones showed calcium phosphate. Tonsillolith was diagnosed based on the findings. Computed tomography showed no recurrence of the tonsillolith at 1‐year follow‐up.

**FIGURE 1:**
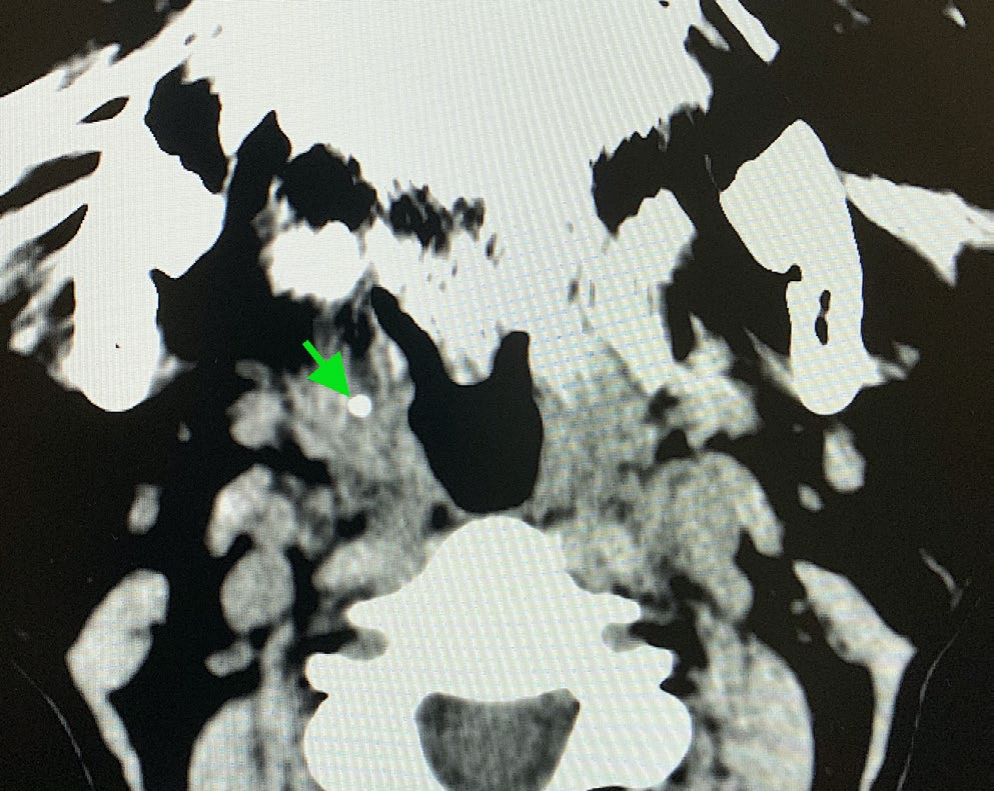
Computed tomography revealed a dense calcified mass in the right palatine tonsil (arrow)

**FIGURE 2:**
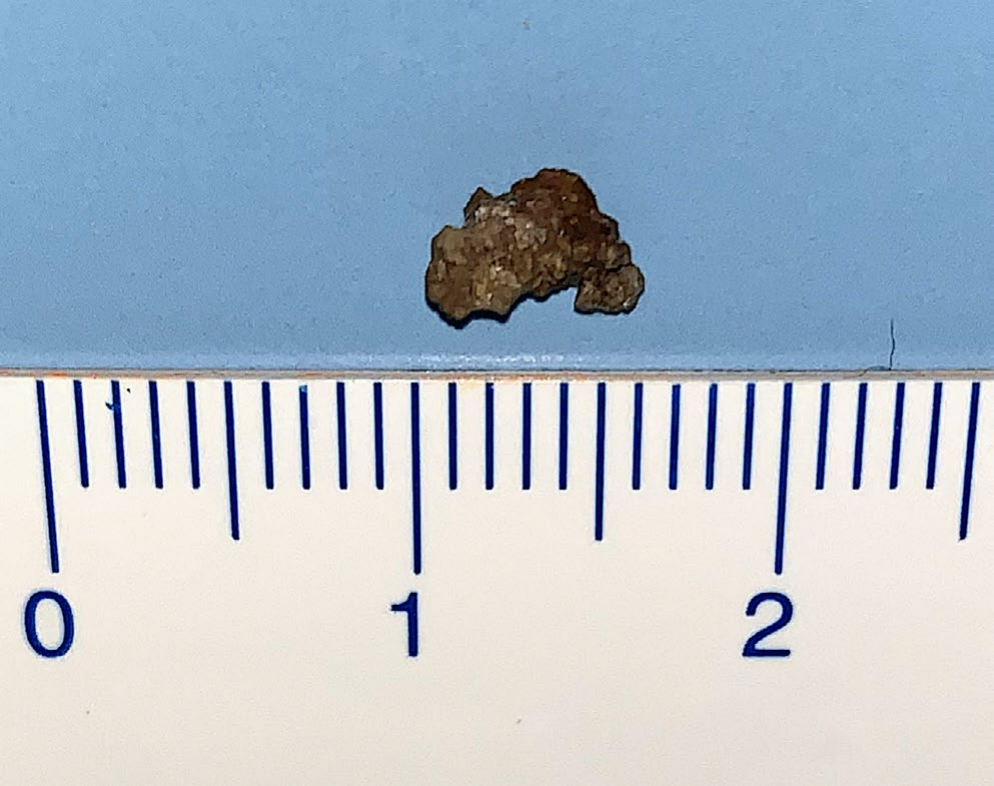
Tonsil stone. 6 mm × 4 mm in size and appeared hard, with an irregular surface

Small tonsilloliths are usually asymptomatic, whereas large tonsilloliths are sometimes associated with recurrent odynophagia^1^ and may cause life‐threatening symptoms such as dyspnea, esophageal perforation, and mediastinitis.^2^ Tonsilloliths are usually removed by enucleation or curettage under local anesthesia, and larger lesions may require local excision and tonsillectomy.^1^ The differential diagnosis of tonsilloliths includes foreign body, calcified granuloma, malignancy, or elongated styloid process.

## CONFLICT OF INTEREST

None declared.

## AUTHOR CONTRIBUTIONS

KS: cared for the patient and wrote the report. KS and MI: read and approved the final version of the report. All authors had access to the data and a role in writing the manuscript.

## INFORMED CONSENT

We have obtained the consent of the patient for publication.

## Data Availability

Data sharing was not applicable to this article as no datasets were generated or analyzed during the current study.
